# Why do some participants in colorectal cancer screening choose not to undergo colonoscopy following a positive test result? A qualitative study

**DOI:** 10.1080/02813432.2018.1487520

**Published:** 2018-09-21

**Authors:** Anne Katrine Lykke Bie, John Brodersen

**Affiliations:** a Anne Katrine Lykke Bie medical student at the university of Copenhagen., Centre of Research & Education in General Practice;; b Centre of Research & Education in General Practice, Primary Health Care Research Unit, Zealand Region

**Keywords:** Colorectal neoplasms, mass screening, cancer screening test, colonoscopy, qualitative research

## Abstract

**Objective:** Our aim was to investigate why participants opted out of colonoscopy following a positive screening result for colorectal cancer.

**Design:** Semi-structured, qualitative, single interviews. We audio-recorded and transcribed all interviews verbatim and used Strauss and Corbin’s concept of open, axial, and selective coding to identify the main categories shared across all interviews. These formed the basis of our findings.

**Setting:** A Danish national colorectal cancer screening programme.

**Subjects:** Single interviews with 13 participants who declined to have a colonoscopy.

**Main outcome measures:** Reasons to decline colonoscopy after positive screening test.

**Results:** Participants gave 42 different reasons for deciding not to have a colonoscopy and we coded them into nine main categories; Practical barriers, Discomfort of the examination, Personal integrity, Multimorbidity, Feeling healthy, Not having the energy, Belief that cancer is not present, Risk of complications, and Distrust in the accuracy of the iFOBT.

**Conclusions:** Our findings suggest that some practical barriers could be quite easily addressed, by offering the participants alternative management and procdures.

**Implications:** Further research is needed to examine how widely our findings are represented in the general population, and how general practitioners should consult with patients who have opted out of colonoscopy, despite a positive screening result.

**Key points**
  Some screening participants are reluctant to proceed with further diagnostic tests for colorectal cancer following a positive screening result.
  • Interviews with people, who had refused a follow-up colonoscopy, discovered nine categories (42 reasons) of reasons for refusal.
  • Reluctance can be addressed by offering support with pre-procedure preparations and alternatives to colonoscopy.
  • General practitioners face ethical dilemmas and challenges, when patients at risk of colorectal cancer decline to proceed with screening.

## Introduction

Medical screening has grown in popularity, especially in socioeconomically developed countries, and during the last three decades the Nordic countries have started to implement mass screening programmes for breast, cervical, and colorectal cancers [1,2]. National colorectal cancer (CRC) screening programmes have been implemented in Denmark and Finland. In Sweden they are available on a county basis, and in Iceland and Norway preparations have been made, but national screening programmes have yet to be implemented.

CRC is the third most common type of cancer and it has the third highest mortality rate. In the five Nordic countries there are approximately 18,000 new cases every year [3], which is one of several reasons why the different health authorities try to implement appropriate screening methods. According to two Cochrane reviews of screening with faecal occult blood test (FOBT) for CRC can lead to a relative reduction of CRC-related mortality of 13-16% [4,5]. However, there have been some inquiries as to the validity of these studies [6].

The Danish mass screening programme for CRC was implemented in March 2014, using the immunochemical faecal occult blood test (iFOBT) as the first line of screening. When the programme is fully implemented in 2018, a biennial invitation to screening will be sent to citizens in Denmark aged 50–74 years. If the iFOBT is positive participants are invited to undergo a colonoscopy in a hospital setting, and any identified polyps or tumours will undergo biopsy or removal.

In January 2016 the Danish Health Authority published a report on the first 10 months of the national CRC screening programme: 64% of those invited, participated in the programme and 6.8% of them had a positive iFOBT. In Region Zealand (one of five health regions in Denmark) 62% of invited citizens participated and 7.4% of them received a positive iFOBT result. Of these, 87% accepted an invitation to have a colonoscopy [7]. Similar non-completion rates have been seen in France (12%) and in the UK (15–18%) [8,9].

On a national basis, approximately 6% of participants who had a screen-positive iFOBT were subsequently diagnosed with CRC, which is an incidence about ten times higher than people in the average population aged 50–74 years [10]. Therefore, people who receive a screen-positive iFOBT who choose not to undergo a colonoscopy risk worsening their prognosis because their likelihood of having CRC is already higher. This could bring ethical dilemmas and challenges to the consultation the next time these people visit their general practitioner (GP). A prerequisite for being able to help these citizens is to understand their motivation for declining the colonoscopy.

The aim of this study was to discover the reasons why people do not undergo colonoscopy after a screen-positive iFOBT, and to provide guidance to GPs when they meet these patients in their practice.

## Material and methods

### Selection and recruitment of participants

Approximately 29.000 citizens in Region Zealand participated in the first ten months of the Danish CRC screening programme; 2175 people had a positive iFOBT and 282 of them chose not to undergo colonoscopy [7].

**Figure 1.**
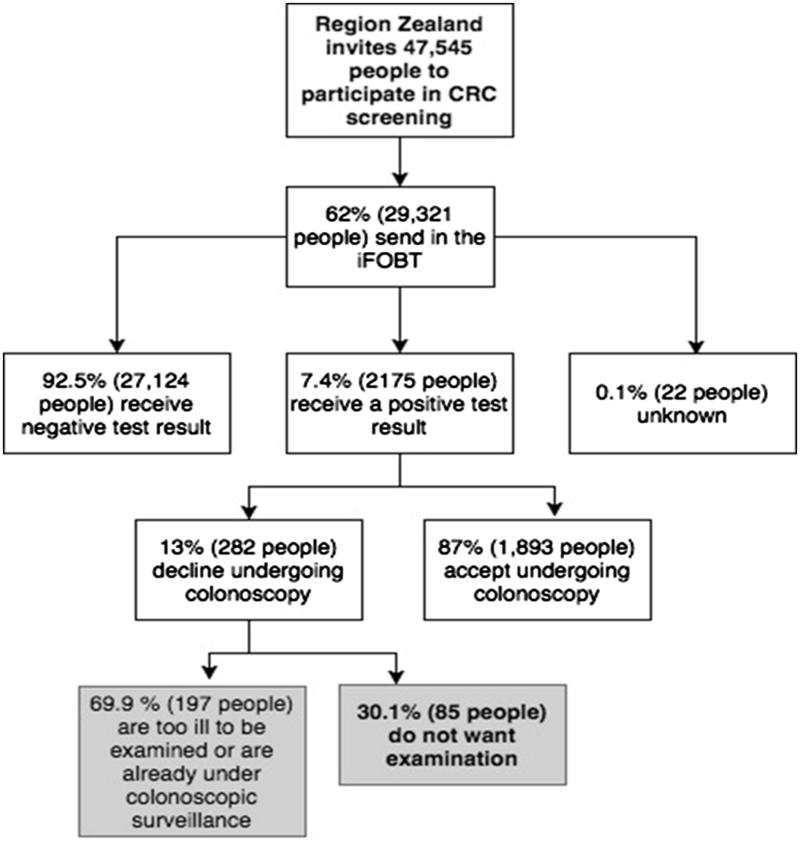
Numbers from a report on the first ten months of the CRC screening, in Region Zealand, one of the five health regions of Denmark [7]. The author of this article however identified the numbers in the boxes marked in grey.

The first author (AKB) had permission to access the free text, written by secretaries at the screening centre, and identified a potential sample of 85 of the 282 people who had chosen to refuse colonoscopy based on the selection of ‘do not want examination’ recorded in the Region Zealand’s CRC screening database. The remaining 197 citizens were diagnosed with an inflammatory bowel diagnosis and were already under colonoscopic surveillance, or suffered from severe multimorbidity, cancer or a terminal illness and were not eligible for colonoscopy (Figure 1), and were excluded from the present study. The free text-entries were anonymized and after isolating the 85 citizens that did not want the colonoscopy, AKB gained access to limited personal data consisting of gender, age, and municipality.

Of the 85 citizens identified, 75 men and women were invited to participate in this study. The potential interviewees were selected by strategic sampling according to age, gender, and municipality to ensure maximal variation. The regional screening centre sent out the invitation-letters, thus ensuring anonymity. In the invitation-letters, the potential interviewees were asked to contact one of the two authors by telephone or e-mail if they wanted to participate in an interview. A reminder was sent to non-responders four weeks after the initial letter of invitation.

13 people responded and were subsequently interviewed.

### Qualitative interviews

AKB is a medical student working with JB who is an experienced researcher in the field of medical screening.

We chose a qualitative research design, where both authors conducted in-depth, single interviews on a semi-structured basis. An interview guide was developed prior to the interviews, and was reviewed and modified after each interview, if needed. This allowed a thorough examination of the informants’ personal attitudes and explanations as to why they had chosen not to undergo a colonoscopy after receiving the result.

In an attempt to decrease the asymmetric power relationship between informant and interviewer, the informants chose the location of the interview. We wanted to ensure the least inconvenience to participants; therefore most interviews took place in their homes or via telephone. One informant chose to be interviewed via e-mail, because he accepted our invitation to participate by writing us a long e-mail where he explained in detail why he had refused colonoscopy. On that basis we replied and asked additional questions, which were answered comprehensively by the informant.

The interviews took place in spring and autumn 2016 and lasted between 20 and 90 minutes each, which enabled an exhaustive exploration of the different reasons for choosing not to undergo colonoscopy. We stopped the interviews when the informants expressed they had told us all reasons to why they declined undergoing colonoscopy. Thus, the length of the interviews varied. The authors wrote contextualizing notes after each interview, which were also digitally recorded, and transcripts were subjected to qualitative content analysis. Thereafter, we deleted the audio recordings.

### Data analysis

To protect the identity of the participants in the study, we only use gender and interview number when we quote them.

We read the transcripts several times and the informants’ experiences and explanations were condensed into units of meaning [11]. We used Strauss and Corbin’s concept of open, axial, and selective coding to identify the main categories shared across all interviews [12]. We translated the Danish quotes we used, into English with help from a bilingual native English speaker.

Any disagreements between the two authors were discussed until consensus was achieved and the interviews were re-audited if necessary to ensure that our findings were firmly grounded in our data. We obtained informed verbal consent from all participants except for the informant we interviewed via email, who gave us a written consent.

## Results

Of the 13 informants, six lived in urban settings and seven in rural settings; six were women and seven were men, and the age range was 51-76 years (see Table 1). One of the 13 informants was incorrectly classified and had in fact received a screen-negative iFOBT, and therefore the interview was stopped. In addition, one informant, who had declined the offer of a colonoscopy based on a previous negative experience in a hospital, later went to his GP, who suggested to him, to have a colonoscopy at a private clinic. Therefore, this informant’s reasons for declining the colonoscopy have been included. The remaining 11 informants did not have any further examinations of their colons following their screen-positive iFOBT. All twelve interviewed informants reported that they were surprised to receive a screen-positive iFOBT and the decision not to undergo the colonoscopy was made post-screening.

**Table 1.** Description of interview participants in random order.

Gender	Age	Education	Career	Residence	Multimorbidity	Interview
M	54	Public school and vocational training	Forest worker	The countryside	Yes	By telephone
M	64	Vocational education	Mechanic	The countryside	Yes	By e-mail
M	75	Vocational education	Carpenter	City	No	By telephone
M	67	Bachelor	Pedagogue	City	No	By telephone
F	51	Public school	Cleaning, early retirement	City	Yes	By telephone
F	73	Public school	Statistical work	The countryside	No	At our offices
F	70	Vocational education	Hairdresser, Nursing home employee	Village	Yes	Home of informant
M	61	Ten years of public school	Corporate chief	City	Yes	By telephone
M	70	Public school	Handyman	The countryside	No	By telephone
F	70	Public school	Odd jobs, early retirement	City	Yes	Home of informant
M	76	Ten years of public school	Public servant, manager of taxation	Village	Yes	By telephone
F	54	Grammar school	Odd jobs, early retirement	City	Yes	Home of informant
F	70	Bachelor	Teacher	Village	No	By telephone

We identified 42 reasons why participants chose not to undergo colonoscopy after a screen-positive iFOBT that could be grouped into nine main categories. Re-reading the transcripts and listening to the audio recordings again validated these categories, which are: Practical barriers, Discomfort, Personal integrity, Multimorbidity, Feeling healthy, Not having the energy, Belief that cancer is not present, Risk of complications, and Distrust in the accuracy of the iFOBT (Table 2).

**Table 2.** Nine categories and 42 reasons why our participants declined colonoscopy following a positive iFOBT.

Found categories and underlying reasons	F1	F2	F3	M4	M5	F6	F7	M8	M9	M10	M11	F12	M13
Practical barriers													
Lack of offer of local anaesthesia						X							
Can not or will not undergo the cleansing alone		X											
Do not want treatment if polyps are detected, so thinks procedure is futile						X				X		X	
Distance to travel										X			
Discomfort													
Have heard about the colonoscopy being uncomfortable	X		X										
Have experienced the colonoscopy as being physically uncomfortable		X		X									
Have experienced the colonoscopy as being psychologically uncomfortable				X									
Have read about the colonoscopy being uncomfortable						X			X				
Have an idea that the colonoscopy is uncomfortable									X				X
Dislike of MOVIPREP			X										
Do not want a big ‘hose’ up their behind													X
Fear of discomfort following the sedative													X
Personal integrity													
Does not like the appearance of his/her “behind”						X							
The colonoscopy is an overstepping of his/her bounds												X	
It is unnatural to put something inside something that is meant to be an ‘exit’												X	
Multimorbidity													
Psychiatric		X	X										
Somatic		X	X		X								X
Feeling healthy													
No reason to provoke something, by touching something healthy	X												
Feeling healthy	X		X				X		X				
Convinced that there is no colon cancer						X							
Don't want to be medicalised							X						
Don't want to go around with a constant worry						X							
No reason to touch anything healthy									X				
Always having had a healthy pelvic area	X												
Not having the energy													
Disease of family members has taken up a big part of life	X												
Own disease has taken up a big part of life		X			X			X					
Lack of energy		X			X								
Belief that cancer is not present													
Probability of it being due to haemorrhoids		X	X		X	X		X		X		X	X
Probability of it being due to a scratch			X				X		X			X	
Lack of family history										X			
Information of other causes of bleeding from doctor	X			X	X								
Lack of physical symptoms			X			X							
Have other pre-existing colon-condition, that can cause bleeding				X		X							
Complications													
Do not want a perforation of the bowel		X											
Lack of trust in the doctors’ abilities to perform the examination without mistakes		X						X					
Trauma from previous experience with the healthcare system								X					
Worried about complications, mentioned in the information leaflet			X					X					
Have heard about people dying from the colonoscopy													X
Distrust in the accuracy of the iFOBT													
Have heard of others who have received false-positive answers			X										
Distrust in the presence of blood, because it is not visible		X	X										
Doctor has said that only a small fraction of people receiving a positive answer are sick					X								
Don't want to participate anymore, since they have never found anything before		X											

Data saturation was achieved after the 13^th^ interview since no new main category was revealed in the last three interviews.

The main categories identified are described in detail below:

### Practical barriers

A range of different practical barriers were mentioned during the interviews. One informant said that he thought the distance between his home and the hospital was too great, and that the procedure was not important enough for him to travel all that way. Other practical barriers were connected to the discomfort of the colonoscopy itself. One informant would have considered it, had there been an offer of local anaesthesia, and another did not want to undergo the pre-colonoscopy bowel preparation:

If it was, that I could go to K⊘ge hospital and be there in the 24 hours I had to be emptied out, and have the stool out of the bowels, and then in the morning, early in the morning, could come in to a screening examination or what it’s called, then I could possibly do it. But, but… they didn’t speak about it down there [K⊘ge hospital, for a pre-examination], when I was down there, and I didn’t dare to propose it, so at that point I was a little afraid. (F2)

A couple of informants also said that if they went for a colonoscopy and CRC was detected, they did not want any treatment. Therefore, in their eyes it was not worth it to undergo colonoscopy.

### Discomfort of the examination

Most of the informants had a clear idea that a colonoscopy is a very uncomfortable examination. Some had no source of information for this idea, while others had heard it from friends and relatives, had read it in the information letter, or had previous experience of colonoscopy. The people who had heard about the procedure from friends or relatives were especially wary of the physical discomfort:

It is because…. It’s all that stuff you have to get up, and they blow into you, some say that it hurts and stuff like that. (F3)

The description of physical discomfort was prevalent in the informants who had read the description of the examination in the letter from the screening service, and the people who had previously undergone a colonoscopy also underlined the psychological discomfort.

When I chose that option, it was because I, some years ago, had a not so good experience of the same examination done by the healthcare system. (…) Well it was the entire… it was the entire way it was executed. You were almost… you were almost put down, in order for it to be performed. (M4)

Some informants who had undergone colonoscopy previously also described the discomfort they had experienced as a result of an insensitive doctor, with regard to their experience of pain during the procedure, as well as their emotional experience.

### Personal integrity

One informant did not want to a colonoscopy because of thoughts about the embarrassment she would experience. This was because she had haemorrhoids and was ashamed of how she looked and she did not want to expose her bottom to a doctor:

And now, I know what it looks like in one end, because I have a haemorrhoid or something. I can, with a rubber glove, get it to look a bit nicer, myself, this damn asshole, right? But eh… No, I couldn’t do it! (F6)

She also described later in the interview how she pictured the doctor wearing something like ‘night-vision goggles’, looking at her bottom, and to her this was an almost unbearable thought, even though she knew that this scenario was not at all likely in reality.

Another woman had difficulty accepting the method of examination and had a feeling of alarm, concerning the rectal examination. She exclaimed that the rectum was meant to be an exit, not an entrance, and she felt an unease about anyone touching something inside her body that she did not feel when talking about breast cancer and mastectomies, or other ‘superficial’ procedures, as she described them:

I mean it *is*… overstepping my bound**s.** I mean, it is the same way with dentists, but then you know how far in they’re going. You don’t know that with an examination like this. And you can also think, what are they gaining by doing it? (F12)

### Multimorbidity

For some informants multimorbidity played an important role in their decision not to undergo colonoscopy. It was not so much facing the specific diagnosis of CRC; rather than the burden of all of their diseases, including psychiatric diseases. Some of our participants experienced anxiety, schizophrenia, and panic-induced claustrophobia. One informant, who was diagnosed with paranoid schizophrenia, said that disease had overwhelmed her during previous medical procedures. She described the experience as a ‘snowstorm’ inside of her head and went on to say:

And I’m also schizophrenic in my thoughts, and that’s really hard for me. And I didn’t think… I don’t think I can do it. If they start operating on me… I’ll kick the bucket. (F2)

Somatic multimorbidities included diarrhoea, sclerosing cholangitis, and diabetes. Two patients with diabetes were especially afraid to take the laxative alone. These patients were unsure what it would do to their blood sugar, and had not felt comfortable asking a doctor about it. One of these patients had asked about the effects of the laxative when she called the secretary at the screening clinic, who had promised that a health professional would call her back. However, that did not happen.

### Feeling healthy

The majority of our informants chose not to undergo colonoscopy because they regarded themselves as healthy. They did not experience any physical symptoms, they had wholesome and active lifestyles, and had a firm belief that they were perfectly healthy. These informants did not see the point in examining somebody who was healthy:

I don’t think there is anything wrong with my colon. So… I mean, if there’s nothing wrong, why should it be examined? (M9)

One informant thought the examination was like ‘using a sledgehammer to crack a nut’, since she was healthy, and the procedure would have the effect of medicalising her more than anything else:

Somehow I think it is like you… it is like medicalising, like me. How can you put it, to take me out there, where I, in no way, feel like I belong, you know? (F7)

The informants, who were convinced there was nothing wrong with them, generally did not want anybody to fiddle with something healthy. Some mentioned that they did not want to provoke something, by having doctors touching healthy tissue. One woman said that she did not want to go around with constant worry created by the procedure, especially not when she felt certain that she was fine.

### Not having the energy

Some informants said they did not have the energy to undergo colonoscopy. More than one informant went on to say that they had either been through a comprehensive course of disease themselves, or experienced it through a spouse. Hence, they felt like they had seen their share of doctors and hospitals and did not feel like they had the energy to enrol on a new course of treatment:

I thought: now again? They can stuff it! I didn’t think anything big of it. I thought: I don’t have the energy for this; I don’t have the energy for this! I am sick and it is my body. (F2)

One informant, who had had a major organ transplant, explained that he was tired after many years of regular visits to the hospitals, and various treatment plans for different complications. When he was offered the colonoscopy, a procedure that he was able to turn down without the certainty of consequences, he said no:

I don’t want to expose myself to that. I have had enough to do with the healthcare system. (M5)

### Belief that cancer is not present

Almost all informants had possible explanations for why there was blood in their stool, other than CRC. These included haemorrhoids, scratches in the rectum, or other non-serious conditions of the colon. Some of the people we interviewed told us they had been reassured by their doctors that the blood could come from elsewhere:

Then when I talked to my doctor in there [at the hospital], then he says yes… but it can be a haemorrhoid, sitting there giving some blood, or it can be a small scratch or a wound or something different. It can be a lot of things. And that doesn’t mean… doesn’t have to… far from has to mean that there is anything there - like he said… (M5)

One man was convinced that he had no risk of developing cancer since there had been no cases of familial cancer. Others were reassured by the lack of physical symptoms:

No, but I am thinking, if there is something there, then you can feel it. (F3)

Another woman told us that she was sure there was nothing wrong, since she had not been able to spot any blood in the stool since receiving a positive result, so her conclusion was that it was probably gone again.

### Risk of complications

Not all informants had read the information material provided in the invitation letter to screening participation, but those who had, said that one reason why they had decided to opt out of the colonoscopy was the risk of suffering from complications during the procedure:

No, and I don’t want holes in my colon either, I saw in the letter that there was a risk of that. (F2)

Another informant had had a very traumatic experience during a previous procedure where he suffered from iatrogenic complications. As soon as he read about the risk of perforation and bleeding in the CRC information leaflet, he was convinced that he was going to turn the offer down:

In the information material it said that during a colonoscopy there could be damage to the colon and bleeding could occur. I don’t know if it makes sense to you, but I am not having any. Especially not, when they are not even sure, anything is wrong. (…) So my conclusion is that I would rather die from colon cancer, than having an incompetent doctor getting lost in my body with some instrument. I have tried that before, and it was no success. (M8)

This informant was clearly scarred from previous experience and for him the risk of suffering from complications was paramount in his decision. Most participants were aware that the risk of complications during the procedure was relatively low, although none had tried to find the exact numbers. For some people any risk was more than enough to support their decision to forego the offer of a colonoscopy.

### Distrust in the accuracy of the iFOBT

Some informants had visited their GPs, or had seen other doctors (e.g. specialists) who had given then information about the iFOBT, and the fact that only a small fraction of the people who tested positive did, in fact, have cancer. Other informants had not discussed the iFOBT with their GPs, and had the misconception that the blood detected by the test is visible. More than one informant expressed their belief that the test result had been incorrect, due to the lack of visible blood in the stool or on the toilet paper:

And I don’t have the impression that, well I haven’t had it examined since, but I have no blood or anything on the [toilet] paper or anything, at all. (F1)

One woman expressed her distrust of the iFOBT giving a true result. She had a sister who had received a positive result previously, but did not have CRC:

And then you heard that many, they told them that they had [blood] in their [stool], and it - and mine had been too. They said she [sister] had it too, but there wasn’t anything there, was there? (…) Then there wasn’t anything after all, was there? That is why I don’t believe them. (F3)

The story of her sister had resulted in her losing faith in the healthcare system. She had also believed that the iFOBT was a direct test for cancer, and had been taken aback to receive the information that she had to undergo a cleansing of the bowel and a colonoscopy. She was the same informant who had tried unsuccessfully to contact the hospital to discuss the laxative treatment with regard to her diabetes, so her faith in the screening programme was somewhat diminished at the time of the interview.

## Discussion

Through 13 interviews, we defined nine main categories that our informants gave for not having a follow-up colonoscopy: Practical barriers, Discomfort, Personal integrity, Multimorbidity, Feeling healthy, Not having the energy, Belief that cancer is not present, Risk of complications, and Distrust in the accuracy of the iFOBT. The nine categories covered 42 different, specific reasons why not to undergo colonoscopy (Table 2).

It was our intention to get as many explanations as possible in order to have a comprehensive understanding of our primary research question. Therefore, it was a strength to choose a semi-structured interview approach, so that participants would feel free to express their thoughts and opinions. We were explicit in our invitation letter about maintaining the informants’ anonymity and that we had used the screening database at Naestved Hospital to send our letters, underlining that we had no access to their personal information until they chose to contact us. Nevertheless, we cannot rule out that some people declined to participate in our study because we used official Region Zealand stationery and postage, and the people who chose to discontinue screening are hard to reach, since they have already opted out of the screening programme.

We have no information on the people who declined our invitation to participate and therefore we do not know if there were any socio-economical differences between them and the participant group. At the outset, we only had access to the gender, age, and municipality of the invitees. There are no statistically significant differences between participants and non-participants in relation to these variables.

A limitation to our study is that since it was retrospective, we cannot exclude the possibility of recall-bias. The informants were all invited to participate in the screening programme in 2014, which means they were asked to remember their thoughts and feelings from one or two years ago when talking to us about their experiences.

We have tried, as far as possible, to uncover all possible reasons for opting out of colonoscopy after receiving a screen-positive result, but we cannot ignore the possibility of participation bias. Previous studies have shown that the people who participate in research projects on screening are typically more psychologically robust than the people who choose not to respond [13,14]. The 42 reasons we identified are therefore perhaps not transferrable to the general population. However since several of the informants in this study were living with severe chronic mental disorders and/or multimorbidity, we suggest that our results are more widely relevant.

To our knowledge all our informants were of Danish ethnicity. Since our sample was guided by the three variables of gender, age, and municipality, we made no exclusions based on ethnicity. Therefore, there is a risk that we have not identified all of the reasons why some people choose to opt out of colonoscopy.

Time and costs are other barriers to nonparticipation in screening and these have been described in other studies [15–19]. However, none of our informants mentioned either time or cost as a reason to decline colonoscopy. In Denmark, access to healthcare is free at the point of use, which would greatly reduce the likelihood of cost impacting a decision to decline. It is uncertain as to why we did not hear about time being an issue. However, one informant told us that he thought the distance to the hospital (about 30 kilometres) was too great, but he talked about not wanting to drive that far as the reason behind his decision.

The authors had some preconceptions before starting the project. We expected to hear about the discomfort of the examination, as this has often been observed in studies investigating barriers to colonoscopy [20]. We also expected to meet some people who talked about the shock of receiving a positive result from screening, as they perceived themselves to be healthy [21]. However, we intentionally did not include specific questions regarding these in the interview guide.

Many studies have examined barriers to colonoscopy before, but often in the context of colonoscopy as primary screening method to detect disease, or as an exploratory examination [20]. So far, we have identified only two studies that examine the decision to discontinue participation in the CRC screening programme, thus dealing with a group of people who are in an intermediary position between knowing they have a positive result from a screening test for a disease, but declining an opportunity to determine if the disease is actually present. One paper is a preliminary study published as a congress abstract [22] and the other is a recent study by Plumb et al. [23], who extracted their data from the screening participants’ medical records (SSP databases), and not via interviews. Plumb et al. only had access to the data that doctors entered in the free text fields of the SSP databases. This could be a limitation in their study since the researchers had no possibility of elaborating the reasons identified in the records. Our interviews gave us that possibility and we could ask our participants to expand on their reasoning. In this way, we may have been able to identify reasons that patients tried to conceal because of feelings of shame, anxiety, or something else. As such, we have identified the category of ‘Personal integrity’ which covers a range of reasons where participants expressed shame or self-consciousness about exposing their bottoms to the colonoscopist; distrust in the test because of what other people had experienced; feeling healthy and not thinking of themselves as having cancer, and refraining from colonoscopy because of psychiatric comorbidity. These reasons have not been reported in the study by Plumb et al. On the other hand, the study design of Plumb et al. enabled them to have access to more explanations since they examined the medical records of 66 people, compared to our 13 interviews. There is a wider variety of sociodemographic information on the patients in the study by Plumb et al. Among other things, some of their informants reported a religious motivation for declining colonoscopy, for example fasting for Ramadan, which was not mentioned by our informants.

We asked our informants if they would consider completing the screening, if they were offered an alternative diagnostic procedure, e.g. CT colonography or capsular endoscopy. Once we had explained what these procedures involved, many of the informants confirmed that they would indeed proceed, confirming findings from previous studies [16,24].

During the interviews, we noted that there were some actions that could be taken in order to improve the experience of CRC screening for participants. Some informants told us that they were afraid to undergo the bowel preparation alone and other studies have shown that the bowel preparation is a barrier to colonoscopy [25,26]. When asked if they would have accepted the colonoscopy if they had been offered admission to a hospital the day before the procedure to have the bowel preparation there, they confirmed that they would.

Another measure that could be taken is the development of a special diet plan for patients with diabetes. In the colonoscopy leaflet participants receive, patients are advised to take only soda, juice, clear soup, etc. on the day before the colonoscopy. It is impossible for patients with diabetes to adhere to this diet without consequences for their blood sugar. Some studies [27] have described that diabetes is a suspected risk factor for developing CRC. This, combined with the fact that approximately 17% of the Danish population between the ages of 50 and 80 years have diabetes [28] makes it seem odd that no diet plan for patients with diabetes is included in the colonoscopy guidance.

This qualitative study has findings that are not yet applicable to the general population. We have described our results and presented some theories within the setting of Region Zealand and further research may unveil more aspects than those we observed in this article, without, however, diminishing those that we have found. In particular, we would be interested in seeing how our findings are represented in the different sociodemographic conditions across all of Denmark and how rare or frequent the different reasons are.

In modern medicine, the involvement of patients in preventive medicine has become immensely popular. But it could be argued that the rise of preventive medicine has transformed risk factors into diseases [29]. This not only changes the challenges facing participants in mass screening programmes, but it also places GPs in a dilemma when faced with patients who have turned down an exploratory colonoscopy after a positive CRC screening test. More research is required into the incidence of CRC among these patients and this could help GPs to manage them in practice.

## Conclusion

We found nine overarching categories, which describe 42 reasons why citizens do not want to proceed to colonoscopy after a positive faecal screening test. We conclude that some practical steps could be taken to reassure these participants, such as having the option to perform bowel cleansing procedures in hospital; developing dietary guidance for bowel cleansing aimed at patients with diabetes; and offering alternative procedures to the participants who dislike the colonoscopy itself and its associated risks.
